# Intraoperative Endoscopy in Transient Adult Jejunojejunal Intussusception

**DOI:** 10.1155/2021/3718089

**Published:** 2021-07-12

**Authors:** Takeshi Okamoto, Hidekazu Suzuki, Katsuyuki Fukuda

**Affiliations:** ^1^Department of Gastroenterology, St. Luke's International Hospital, 9-1 Akashicho, Chuo-ku, Tokyo 104-8560, Japan; ^2^Department of Gastroenterology and Hepatology, Department of Internal Medicine, Tokai University School of Medicine, 143 Shimokasuya, Isehara, Kanagawa 259-1143, Japan

## Abstract

Despite improvements in imaging modalities, causative lead points in adult intussusception may be difficult to diagnose. Such lead points can be malignant, causing recurrence or metastases if left unresected. We describe a case of transient adult jejunojejunal intussusception, in which intraoperative endoscopy was used to confirm the absence of a lead point. A 39-year-old woman with a history of laparoscopic oophorectomy presented with epigastric pain, nausea, and vomiting. Contrast computed tomography revealed jejunojejunal intussusception, with no visible lead point. Spontaneous reduction was confirmed during exploratory laparoscopy. After lysis of adhesions, intraoperative peroral jejunoscopy was performed with the surgeons' assistance. Endoscopy confirmed the absence of tumor, and bowel resection was avoided. No recurrence has been observed during 24 months of follow-up. Intraoperative endoscopy may provide additional reassurance for the absence of a lead point in cases where preoperative enteroscopy cannot be performed and no lead points can be identified on imaging.

## 1. Introduction

While intussusceptions in children are commonly idiopathic, adult intussusceptions (AI) generally have a causative lead point. Enteric intussusceptions, the most common type accounting for about 50% of AIs, have been reported to result from malignant tumors in 22.5% of cases, with metastatic carcinoma being the most common malignant lead point [1]. On the other hand, about 15% of AIs are idiopathic, with no apparent lead point.

One type of AI, classified as idiopathic by some authors, is caused by adhesions resulting from prior surgery [1–3]. Truly idiopathic AI may be caused by mechanisms such as bowel hyperactivity and be transient, causing chronic, intermittent, or nonspecific symptoms [4, 5]. While exploratory laparoscopy with bowel resection remains the most widely accepted treatment for AI, the need for such interventions has recently been brought into question [6, 7]. Almost 5% of AI patients were treated conservatively in a meta-analysis [1]. On the other hand, avoiding surgery may occasionally come at the cost of missing a malignant small bowel tumor, which may be undetected despite recent advances in imaging modalities [8–12]. Such an event may have various devastating results, including recurrence and metastatic disease.

Although not always possible preoperatively, enteroscopy offers an alternative to confirm the absence of a lead point without bowel resection. Herein, we report a case of intraoperative endoscopy performed on a patient with jejunojejunal AI which demonstrated the absence of tumor in the jejunum, providing additional reassurance that bowel resection could be avoided.

## 2. Case Presentation

A 39-year-old woman presented with epigastric pain, intermittent nausea, frequent vomiting, and loss of appetite over the last seven days. She denied weight loss. She could pass gas but had not passed stool over the last four days. She was able to tolerate a full meal the night before presenting to the hospital, but nausea and vomiting had resumed within several hours.

Her medical history was significant only for laparoscopic oophorectomy for a left ovarian cyst almost 20 years prior. She was taking no medications, herbal remedies, or nutritional supplements. She admitted to chronic alcohol abuse with frequent visits to the emergency department due to alcohol intoxication, but had never smoked cigarettes. She denied any recent sexual contact, overseas travel, raw food ingestion, or sick contacts.

Upon presentation, the patient appeared to be in moderate distress. Her respiratory rate was 24 breaths per minute, but vital signs were otherwise stable. She complained of discomfort on palpation of the epigastric region. The upper abdomen was soft but mildly distended. No mass was palpated. Small scars from previous laparoscopy were noted.

**Figure 1:**
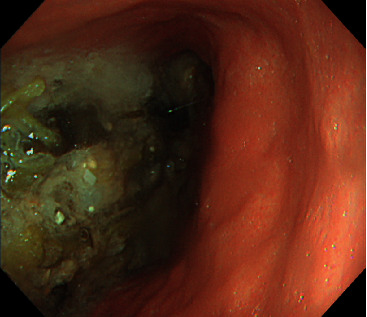
Esophagogastroduodenoscopy performed 20 hours after the patient's last meal revealed significant food residue in the esophagus and stomach, suggesting possible bowel obstruction.

Laboratory results were only remarkable for a mildly elevated C-reactive protein of 0.44 mg/dL. Esophagogastroduodenoscopy (EGD) performed 20 hours after the patient's last meal revealed a mildly distended stomach with significant food residue in the esophagus and stomach (Figure 1). The patient vomited copious food residue during the procedure, precluding a thorough examination for fear of aspiration. No gross abnormalities were found up to the third part of the duodenum. Food residue and intraluminal air were suctioned to the extent possible at the end of the examination.

**Figure 2:**
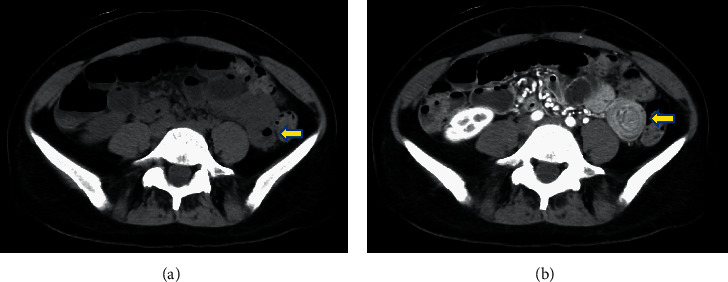
(a) Computed tomography (CT) without contract was largely unremarkable, with no visible signs of tumor or bowel obstruction in the jejunum (arrow). (b) CT with contract taken several minutes later revealed a bowel-in-bowel configuration with invaginated mesentery, consistent with jejunojejunal intussusception (arrow). No mass was visualized.

Despite the patient's ability to pass gas, an emergency computed tomography (CT) scan was conducted to rule out small bowel obstruction. CT without contract was largely unremarkable, with no visible signs of tumor or bowel obstruction (Figure 2(a)). However, CT with contrast taken several minutes later revealed the “target sign,” a bowel-in-bowel configuration measuring 7 cm in the proximal jejunum with invaginated mesentery (Figure 2(b)). No lead point was identified. No proximal distension was observed, most likely as a result of vomiting and suction during EGD. The patient was diagnosed with jejunojejunal intussusception, most likely of a transient nature.

While the possibility of spontaneous reduction was explained, the patient wished to undergo exploratory laparoscopy due to the severity of her symptoms. The surgeons also agreed to exploratory laparoscopy in light of the severe obstructive symptoms, surgical history with possible adhesions, and possible recurrence if left untreated. Consent for intraoperative endoscopy was also obtained to evaluate the jejunum for a possible lead point, as the patient was not in a condition to undergo preoperative enteroscopy.

**Figure 3:**
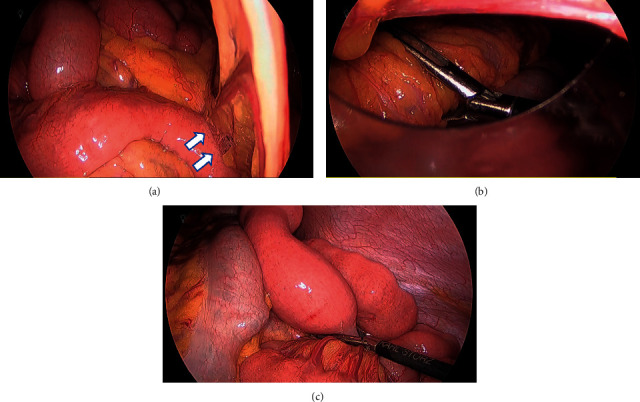
(a) Adhesions from previous laparoscopic oophorectomy observed near a port placed in the right lower quadrant (arrows). (b) The surgeons used laparoscopic grasping forceps to apply gentle pressure to the stomach to facilitate scope insertion during intraoperative peroral jejunoscopy. (c) When the endoscope reached the jejunum, the laparoscopic camera was pointed distal to the suspected location of the reduced intussusception to signal the desired destination for endoscopic viewing. Forceps were also gently placed at this location to facilitate insufflation.

Exploratory laparoscopy was performed the next day. Severe jejunal hyperactivity was observed intermittently throughout the laparoscopy. However, no intussusception was observed, suggesting spontaneous reduction. Tumors and segmental edema in the proximal jejunum were also notably absent. Adhesions from previous laparoscopic oophorectomy were observed near a port placed in the right lower quadrant (Figure 3(a)). While adhesiotomy was performed, the adhesions were distant from the proximal jejunum and appeared as an unlikely cause of intussusception.

**Figure 4:**
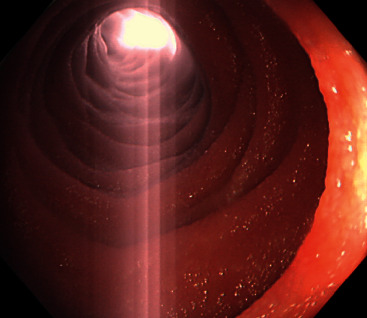
Light from the laparoscopic camera showing through the jejunal wall confirmed passage of the endoscope beyond the site of intussusception.

Intraoperative peroral jejunoscopy was performed with a long colonoscope (PCF-H290L, Olympus Corp., Tokyo, Japan) and carbon dioxide insufflation. A thorough laparoscopic exploration was completed prior to commencing intraoperative endoscopy, as endoscopic insufflation would hinder the laparoscopic view. The surgeons used laparoscopic grasping forceps to apply gentle pressure to the stomach to facilitate scope insertion (Figure 3(b)). When the endoscope reached the jejunum, the laparoscopic camera was pointed to a location in the jejunum believed to be distal to the reduced intussusception. Forceps were also gently placed at this location to facilitate insufflation (Figure 3(c)). The endoscope was inserted until light from the laparoscopic camera was visualized, confirming the absence of tumors or other lesions which may serve as a lead point for intussusception (Figure 4). Short bowel resection was therefore not performed. The patient was diagnosed with transient jejunojejunal intussusception, more likely associated with chronic alcoholism rather than adhesions from previous surgery.

The patient experienced complete resolution of her symptoms after the surgery. The postsurgical course was uneventful and the patient was discharged two days later, with instructions to stop drinking alcohol. No recurrence was observed during 24 months of follow-up.

## 3. Discussion

AI presents two problems for the patient: symptoms relating to bowel obstruction and a potentially malignant lead point.

AI can be difficult to diagnose due to its rarity and the chronic, nonspecific nature of its symptoms [1, 4]. In general, CT has a sensitivity of 83% in diagnosing the etiology of small bowel obstruction and of 82% in diagnosing small bowel tumors as the cause [13]. Helical CT-enteroclysis had particularly high pooled sensitivity (92.8%) and specificity (99.2%) for small bowel tumors in a meta-analysis [9]. On the other hand, only 52% of enteric AIs were recognized preoperatively in a study of 44 patients [7]. Another study of 318 patients found that AI patients presented with symptoms of complete and partial bowel obstruction in only 27% and 15% of cases, respectively [8]. CT failed to identify lead points in reports of jejunal AI caused by heterotopic gastric mucosa, laterally spreading tumor, and gastrointestinal stromal tumor [10–12].

While symptoms can be relieved by reduction or bowel resection, recurrence has been reported in about 6.5% of all AI cases [1]. This figure may be higher in enteric AI, as one report found recurrences in 21 of 230 enteric AI patients (9.1%) [8]. All recurrences occurred at the site of the initial AI and 63% required surgery. Recurrence occurs frequently in celiac disease, Crohn's disease, and polyposis syndromes such as Peutz–Jeghers syndrome due to their multifocal involvement [14–16]. Recurrence has also been reported in idiopathic enteric AI [17].

Transient intussusceptions with spontaneous reduction are very common in children as they are generally idiopathic in nature, particularly when the length of the intussusception is less than 3 cm [18, 19]. Similarly, intussusception length of less than 3.5 cm is an independent predictor for self-limiting AI [20]. The diagnosis of AI on CT may be more common than once believed, in part due to improvements in imaging technology. In a study of 37 CT diagnoses of AI, 31 were cared conservatively and none required surgery during a mean follow-up of 5.2 months [20]. Although we suspected transient AI based on the discrepancy between the plain and contrast CT scans in our case, exploratory laparoscopy was believed to be indicated due to the length of the intussusception (7 cm), severity of symptoms, possibility of recurrence, and the wishes of the patient.

As the adhesions observed during laparoscopy were distant from the site of intussusception, we suspect that bowel hyperactivity due to chronic alcoholism played a role in the pathogenesis of AI in our case. The effects of alcohol on small bowel motility depend on the alcohol concentration of consumed beverages and chronicity of alcohol use, with chronic consumption of large doses of alcohol accelerating small bowel transit [21, 22]. There is only one report of small bowel intussusception in a patient with chronic alcoholism, although various other factors such as malnutrition and brown bowel syndrome were also present in that case [23]. The role of alcohol use in transient AI has not been studied; the history of alcohol use is generally missing from case reports on AI. While alcohol is also associated with diarrhea due to inhibited water and sodium absorption as well as mucosal injury in the duodenum and upper jejunum, none of these findings were observed in our case [24]. The use of scopolamine during preoperative EGD may have temporarily reduced bowel hyperactivity, contributing to temporary resolution of AI before the CT scan.

Both antegrade enteroscopy and retrograde enteroscopy have been used to diagnose various types of lead points including gastrointestinal stromal tumor, inverted Meckel's diverticulum, inflammatory fibroid polyp, Peutz–Jeghers syndrome, heterotopic pancreatic mass, malignant melanoma, and mass-forming fibrogranulation from a healed ulcer [25–31]. Preoperative enteroscopy can also identify signs of bowel ischemia, which occurs in about 15% of cases [1]. Furthermore, the balloon used in double-balloon enteroscopy has also been shown to be useful in achieving reduction of the intussusception [25, 28].

Endoscopy has been used in various surgical procedures such as laparoscopic endoscopic cooperative surgery, confirmation of anastomoses during gastrointestinal surgery, and in exploratory laparotomy for obscure gastrointestinal bleeding [32–34]. Intraoperative endoscopy can be performed via the peroral route, the transanal route, and enterotomies, achieving total small bowel visualization in 57–100% of cases [34]. Advantages over single-balloon or double-balloon enteroscopy include one-stage intervention when a tumorous lead point is discovered, the surgeon;s manual assistance during scope insertion, the use of enterotomies, and performance under general anesthesia in the operating theater [35, 36]. However, single-balloon enteroscopy and double-balloon enteroscopy have reduced the need for intraoperative endoscopy for diagnostic purposes, which is now only used when preoperative enteroscopy cannot be performed or when the diagnosis remains in question [37]. In our case, preoperative enteroscopy was not realistic without deep sedation and intubation, as copious vomiting, strong gag reflexes, and body movement were observed throughout the preoperative EGD. As the site of intussusception could be reached with a long colonoscope, we avoided the use of intraoperative double-balloon enteroscopy which would require additional time, cost, and preparation.

Reports of intraoperative endoscopy in the setting of AI are mainly limited to Peutz–Jeghers syndrome [38, 39]. There are isolated reports of intraoperative endoscopy for AI due to Meckel's diverticuli, cavernous hemangiomas, and duodenal pseudopolyps [40–42]. To the extent of our search, there are no reports in the literature on intraoperative endoscopy in suspected transient AI. However, it is difficult to be confident that there is no lead point, as CT scans can give false-negative results. Patients are often unable to tolerate preoperative enteroscopy, and capsule endoscopy is contraindicated in AI presenting with small bowel obstruction.

Insertion of the long colonoscope could be achieved without fluoroscopy with the surgeons' assistance. Although surgeons' hands in a laparotomy would be ideal, grasping forceps during laparoscopy provided helpful resistance during scope insertion in our case. The camera light clearly showed the planned destination for the enteroscopy in an otherwise uniform bowel. Intubation under general anesthesia allowed for a painless procedure with no risk of aspiration.

In conclusion, we report a case of transient jejunojejunal AI in which the absence of tumor was confirmed by intraoperative endoscopy. Intraoperative endoscopy may be helpful to assess the need for small bowel resection when no lead point can be identified on preoperative imaging and preoperative enteroscopy cannot be performed.

## Data Availability

The data used to support the findings of this study are available from the corresponding author upon request.
